# Relationship between social capital and heroin use behaviors among patients in methadone maintenance treatment in Sichuan Province, China: A cross-sectional study

**DOI:** 10.1097/MD.0000000000019963

**Published:** 2020-06-12

**Authors:** Shifan Yang, Bo Gao, Jing Gu, Yi Gong, Bin Yu, Jiayu Han, Peijie Dong, Peng Jia, Shujuan Yang

**Affiliations:** a West China School of Public Health and West China Fourth Hospital, Sichuan University, Chengdu; b School of Public Health, Sun Yat-Sen University, Guangzhou; c Center for AIDS/STD Control and Prevention, Sichuan Center for Disease Control and Prevention, Chengdu, China; d International Initiative on Spatial Lifecourse Epidemiology (ISLE); e Faculty of Geo-information Science and Earth Observation, University of Twente, Enschede, The Netherlands.

**Keywords:** China, covertly using heroin, intervention, risk factors, social capital

## Abstract

Covertly using heroin during methadone maintenance treatment (MMT) is very common among heroin-dependent patients, which has posed threats to the physical health of heroin-dependent patients and social safety. Covertly using heroin may be influenced by many factors, especially social capital. Therefore, we aimed to investigate the relationship between behaviors of covertly using heroin during MMT and social capital heroin-dependent patients in Sichuan Province, China. A cross-sectional study was conducted between October and November 2018, with a total of 581 heroin-dependent patients participating in the study. In addition to socio-demographic characteristics and heroin use related behaviors, the questionnaire also included the measures of social capital: social network (SN), social support (SP), community participation (CP) and social trust (ST). Multivariate logistic regression analyses were used to estimate the association between different measures of social capital and heroin use. The prevalence of covertly using heroin of heroin during MMT was 31.0% among our participants in the 6 months before the study. After adjusting for socio-demographic factors and heroin-use related variables, SN (OR = 0.85, 95% CI: 0.76–0.95), SP (OR = 0.89, 95% CI: 0.83–0.95), and ST (OR = 0.88, 95% CI: 0.81–0.95) were significantly associated with heroin use. Results suggest that social capital may have a protective effect on behavior of covertly using heroin during MMT, which should be consider in the interventions for heroin-dependent patients, in order to reduce the incidence of heroin use during MMT as well as improve the compliance of MMT.

## 1 Introduction

Drug dependence is one of the major problems which the world face today.^[1]^ The United Nations Office on Drugs and Crimes (UNODC) showed that about 275 million people around the world have used drug at least once by 2018,^[2]^ and 29.5 million people who used drug have problems with drug dependence and 10 million people are incapacitated by drug. Drug dependence not only causes serious damage to individual physical health,^[3,4]^ mental health^[5]^ and social functions,^[6]^ but also plays a positive role in the increase of crime rate^[7]^ and the spread of AIDS.^[8]^ A multistage systematic review^[9]^ showed, 17.8% of people who inject drugs were living with HIV, 52.3% were HCV-antibody positive, and 9.0% were HBV surface antigen positive in the world.

Since 2004, China, one of the countries most affected by the problem of drug dependence, has been implementing methadone maintenance treatment (MMT) for patients with opioid (e.g., heroin) dependence.^[10,11]^ MMT has achieved remarkable achievements in reducing the use of heroin, reducing the spread of blood-borne diseases such as AIDS,^[12]^ and improving the quality of life during recent years.^[13]^ A study^[14]^ showed that 102 heroin addicts reduced their daily heroin use from baseline to 0.0 after 6 months of MMT. No participant seroconverted to HIV and HCV positivity during the 6 months of MMT.

However, repeated client drop-out and re-enrollment cycles is very common in China.^[15]^ Sichuan, as one of the most seriously drug-using provinces in China, had a large number of heroin-dependent patients, which poses challenges for the development of MMT, such as low treatment retention rate and low compliance. Low MMT compliance not only affects the treatment effect of heroin-dependent patients’ MMT, but also increases the risk of relapse of heroin, resulting in damage to the health, increasing the crime rate and pose a threat to social stability.^[16]^

Therefore, it is necessary to improve methadone compliance of heroin-dependent patients. MMT compliance can be measured by covertly using heroin during MMT.^[15,17]^ Because the psychological craving for heroin is difficult to eliminate in a short time, heroin-dependent patients covertly use heroin during MMT.^[18,19]^ Meanwhile, low doses of methadone, social and family discrimination can all affect heroin use during MMT.^[10,17–20]^ Most of these factors are related to social capital.

The theory of social capital is one of the research hotspots in politics, economy and health in recent years. Social capital is a resource existing in the social structure, which is embodied in personal relationship and social network, meanwhile, these characteristics can improve the efficiency of society by promoting coordinated action.^[21]^ Social capital can be divided into micro-social capital which emphasizes the status of individuals in the social structure, medium- social capital which takes the organizational level as the research background, and macro-social capital which pays more attention to.^[22]^ Social network, social support, community participation and social trust are important measurement indexes in social capital research.^[23]^ A multi-level study on harmful alcohol drinking behavior of Chinese residents shows that social capital can influence the occurrence of harmful alcohol drinking behavior of Chinese residents.^[24]^ Under the influence of Chinese alcohol drinking culture, social capital adjustment might help to reduce the occurrence of harmful alcohol drinking,^[24]^ which suggested that social capital can better predict the occurrence of healthy behaviors.

Social capital can provide not only basis for making decisions of healthy behaviors, but also ideas for behavioral intervention and health education.^[25]^ People have obvious sociality, the occurrence of behavior is often closely related to social structure and cognition. Social capital is an important resource existing in the relationship of social structure, which is one of the important predictor of behavior occurrence.^[25]^ In a study of health interventions in Maputo, Mozambique, social capital played a key role in the success of health management decisions, and low levels of social capital were associated with the failure of collective action.^[26]^ Yusuf Ransome et al^[27]^ reported that social capital was closely related to regional differences in AIDS patients’ treatment, and can be used to modify the policy and provide targeted intervention measures for AIDS patients, which would effectively reduce regional differences.

At present, studies on the application of social capital to MMT are relatively rare. The research did not fully considered the social capital, but only part of it such as social support and social network,^[28–30]^ especially the behavior of heroin use during MMT. At the same time, as China is a country with a history and tradition of “relational society”, behaviors of heroin-dependent patients are might be closely related to the “relational society”. Having better “popularity” means having more social capital.^[31]^ Therefore, we conducted a cross-sectional study on the relationship between heroin-dependent patients’ behavior during MMT and social capital, and based on the results, provided some ideas on how to use social capital to develop intervention methods for heroin-dependent patients.

## 2 Materials and methods

### 2.1 Ethics statement

All participants were voluntary joined in our study and signed informed consent forms before survey. The design and implementation of this study protocol was approved by the Ethics Committee of the West China School of Public Health and West China Fourth Hospital, Sichuan University.

### 2.2 Study population and sampling

A cross-sectional study was conducted in Sichuan province from October to November in 2018. The inclusion criteria were:

1) at least 18 years old at the time of joining MMT,
2) meeting the criteria of the national guidelines for the treatment of addictive patients, and
3) participated in the community-based MMT clinics where they lived

Those who had severe cognitive and mental disorders (schizophrenia and bipolar disorder) were excluded from the study.

A stratified multi-stage sampling design was adopted. Currently, there are only 49 MMT clinics in 19 districts of Sichuan province. Because of the household registration system, heroin-dependent patients must participate MMT at local MMT clinics. The population mobility of heroin-dependent patients is low and the population is concentrated, so the cumulative number of patients in MMT clinic can reflect the prevalence of heroin dependence in the clinic. Therefore, first of all, we divided 49 MMT clinics according to the cumulative number of patients, and selected 19 clinics with the cumulative number of patients above 1000. Secondly, we grouped MMT clinics into 4 groups according to the geographical location, and then randomly selected 5 MMT clinics as our research sites.This method was also used in the study of Yu, B. et al. ^[46]^.Finally, in each selected MMT clinics, we invited patients who met the inclusion criteria to join the study.

The sample size was calculated based on 
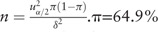
 (64.9% of heroin – dependent patients registered in the treatment system were treated at MMT clinics in Sichuan province, 2016), δ = 0.05, α = 0.05, 
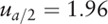
, and the sample size was calculated to be 350. However, due to the large sampling error of the cluster, the sample size was usually 1.5 times of the sample size of the pure random sample, so the sample size was about 525.

### 2.3 Data collection

We conducted an anonymous questionnaire survey on heroin- dependence patients by face-to-face survey. All investigators received two days of investigative training. At the beginning of the survey, the investigators read informed consent forms to the participants and then conducted the survey with their consent. Then the investigators read the questionnaire and recorded the participants’ answers. During the survey, the investigator and the participant sat at the same table, and the investigator's seat was always at a 45° Angle to the participant. The environment for filling in the questionnaire was quiet, closed and alone. A gift (about 15 RMB or 2 USD) was given to participants upon completion of the interview) for their time spent.

Of the 634 participants, 581 (91.6%) provided written informed consent and completed the interview, and 53 (8.4%) declined to participate in the study due to lack of time (e.g., they had to rush to work) and/or other reasons (e.g., they disliked our gifts). The median time for 581 participants to answer a questionnaire was 10 minutes (6–13 minutes).

### 2.4 Design of the questionnaire

A panel consisting of 2 epidemiologists, one health psychologist, and 5 methadone replacement therapy patients was formed to design the questionnaire. The questionnaire was tested among four other methadone replacement therapy patients. Based on their feedback, discussion was made by the panel to finalize the questionnaire.

### 2.5 Measurement

A three-part questionnaire was used to perform the investigation. The first part covered socio-demographic characteristics, the second behaviors of heroin use, and the third social capital.

#### 2.5.1 Participant profiles

Socio-demographic status was considered to include gender, age, nationality, local official registration (also called *hukou*), highest education level attained, and inhabiting information.

#### 2.5.2 Behaviors of heroin use

The researchers studied the participants’ history of heroin use, including when they used the drug and how they used it (e.g., if they used it intravenously). The questionnaire also included a survey of the participants’ other drug use. The history of drug dependence among participants was reviewed, including history of forced drug withdrawal and voluntary drug withdrawal. The researchers surveyed participants about their heroin use during the MMT and divided them into those who had covertly used heroin in the past 6 months and those who had not covertly used heroin in the past 6 months.

#### 2.5.3 Social capital

The social capital questionnaire included four dimensions,^[23]^ with a total of 17 variables quantifying participants’ relationships with family, relatives, friends, and communities. The first dimension was social networks (SN) with 6 variables, measuring the relationships and networks with family members, friends, and neighbors (e.g., you get on well with your family recently). The second dimension was social support (SP) with four variables, including available support from family, friends, and neighbors (e.g., your family always support you on emotion or financial in the past year). The third dimension was community participation (CP) with 2 variables, including participation in neighborhood activities and sense of belonging (e.g., in the past year, you have usually participated in organization activities). The last dimension was social trust (ST) with 5 variables, concerning trust and equity (e.g., you have a lot of trust in medical institutions like hospitals and CDC). The answers consisted of 5-point Likert scales. The response categories were: 1 = *strongly disagree*, 5 = *strongly agree*. Using exploratory factor analysis, 4 factors with characteristic roots greater than 1 were extracted from 17 items, and the cumulative variance contribution rate was 56.81%. The internal reliability (Cronbach alpha) of the scales formed ranged from 0.71 to 0.92 (see Table 2 for individual items).

### 2.6 Statistical analysis

The purpose of our study was to explore the relationship between various dimensions of social capital (social support, social network, community participation, and social trust) and the behavior of participants used heroin covertly. Therefore, the multivariate analysis model to explain the impact of social capital on the behavior of covertly using heroin was the most important goal of our data analysis. In order to adjust the results of the multivariate analysis model, the results of univariate analysis of covertly using heroin and background factors such as socio-demographic variables and potential risk factors were secondary but necessary.

Descriptive statistics were used to summarize characteristics of the study respondents by expressing the results as mean ± standard deviation (SD) or percentage. The prevalence of covertly using heroin, as well as their respective 95% confidence intervals (CI) were presented. When considering covertly using heroin in the last 6 months before the study as dependent variables, univariate odds ratios (ORu) of background variables were identified first. Then crude odds ratios (ORs) and 95% confidence intervals (95% CIs) were calculated with three logistic regression analyses to measure associations between social capital and covertly using heroin in the past 6 months. In the first logistic regression analysis model (model 1), we calculated the crude OR (95% CI) for the relationship of social capital with covertly using heroin. In model 2, the OR (95% CI) was adjusted by controlling for socio-demographic variables. In model 3, the OR (95% CI) was adjusted by controlling for both socio-demographic variables and potential risk factors. The socio-demographic variables and potential risk factors entered into the multivariate analysis model were found to be significant in the respective univariate analysis. Variables with *P* > .10 in univariate analysis were not included in multivariate analysis model. The same analytical methods have been used in a number of published studies.^[32,33]^ SPSS version 20.0 for Windows (SPSS Inc., Chicago, IL) was used for data analysis, with *P* values <.05 taken as statistically significant.

## 3 Results

### 3.1 Socio-demographic characteristics of the participants

In this study, 75.6% of participants were male, and 48.9% were between 40 and 50 years old. 35.3% of participants had attended senior high school or above. Of the participants, 64.7% of them were living with their family. Almost all the participants were Han Chinese (98.3%), registered permanent residence in rural areas (90.7%) (Table 1).

**Table 1:**
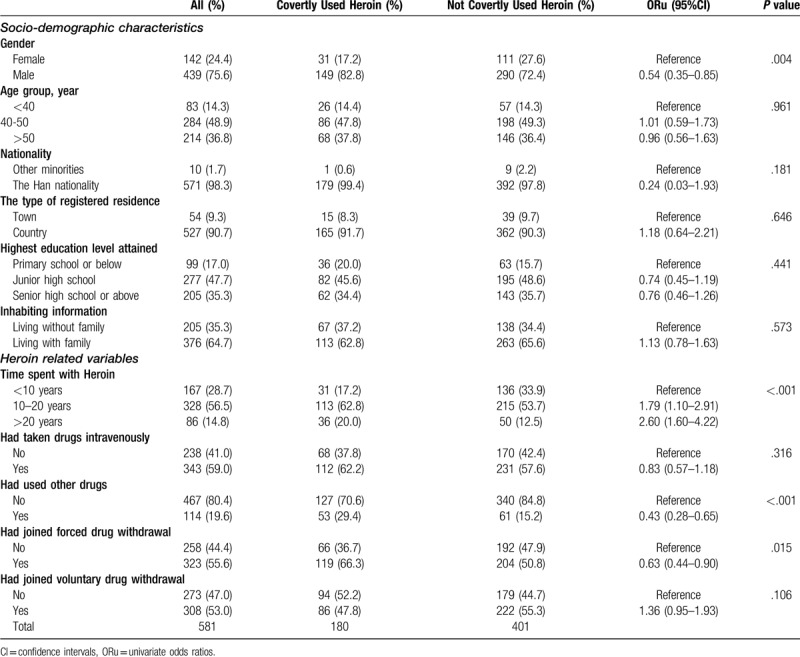
Background characteristics and the behavior of drug use of the participants.

The gender of male was negatively correlated with heroin use in the past 6 months (OR = 0.54, 95% CI: 0.35–0.85). However, the relationship between covertly using heroin and age groups, nationality, the type of registered residence, highest education level attained and inhabiting information were not statistically significant.

### 3.2 Characteristics of heroin use behavior of the participants

Heroin use behavior can be divided into 2 parts: before MMT and after MMT.

Covertly using heroin during the MMT in the past 6 months was used as an indicator of heroin use behavior after participation in the MMT. The 31.0% covertly used Heroin in the last 6 months when they were participating in the MMT (Table 1).

Before participating in the MMT, over half of participants had been on drugs for 10 to 20 years (56.5%), had taken drugs intravenously (59.0%), had not used any drugs other than Heroin (80.4%), had participated in forced drug withdrawal (55.6%) and voluntary drug withdrawal (53.0%). By using univariate analysis, there was a positive correlation between heroin use for 10 to 20 years and heroin use during MMT in the last 6 months (OR = 1.79, 95% CI: 1.10–2.91), as well as, heroin use for at least 20 years (OR = 2.60, 95% CI: 1.60–4.22). Had used other drugs (OR = 0.43, 95% CI: 0.28–0.65) and had joined forced drug withdrawal (OR = 0.63, 95% CI: 0.44–0.90) were negatively correlated with heroin use in the past 6 months. The relationship between covertly using heroin and had taken drugs intravenously and had joined voluntary detoxification were not statistically significant.

### 3.3 Social capital characteristics of the participants

The responses of heroin-dependent patients who have covertly used heroin in the past 6 months to the social capital items are summarized in Table 2. In terms of SN, only 18.9% of participants socialized with closer people except their family in the past month. The 41.7% of participants had a lot of trust in people they interact with on a daily basis. In terms of SP, the frequency of support for participants greatly varies between family members (58.9%) and other people (13.3%). Regarding CP, participants had low involvement in the organization (7.2%) and received little support from the organization (5.6%). Finally, with respect to ST, participants’ trust in medical institutions (87.2%) was higher than that in social organizations (55.0%) and government departments (57.8%). More than half of the participants had a high sense of social justice (83.9%-88.9%).

**Table 2:**
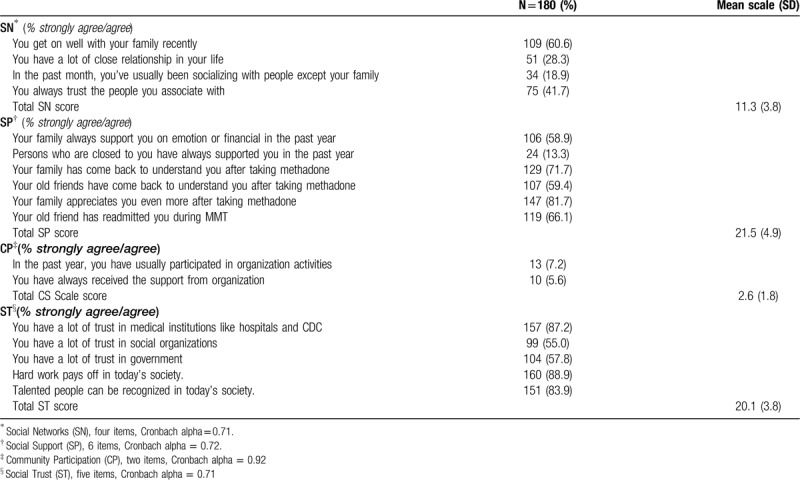
The social capital characteristics of heroin-dependent patients covertly used heroin in the past 6 months (n = 180).

### 3.4 Associations social capital and covertly using heroin in the last 6 months

The associations between social capital and covertly using heroin in the past 6 months in the logistic regression models are summarized in Table 3. SN, SP, and ST were significantly associated with covertly using heroin. In model 1, higher SN was associated with covertly using heroin in the past 6 months (OR = 0.85, 95% CI: 0.76–0.95), and the same was also seen for SP (OR = 0.89, 95% CI: 0.83–0.95) and ST (OR = 0.88, 95% CI: 0.81–0.95). After adjusting for socio-demographic variables and risk factors, SN, SP and ST were still significantly associated with covertly using heroin in the past 6 months in models 2 and 3. However, the association between CP and covertly using heroin not significant in any of the logistic regression models (OR = 0.91, 95% CI: 0.72–1.16).

**Table 3:**
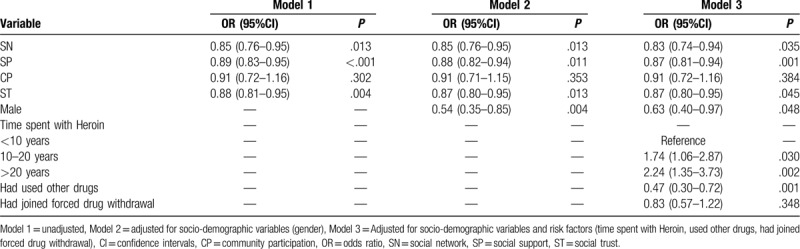
Relationships of SN, SP, CP, ST to the covertly heroin use in the past 6 months (n = 180).

## 4 Discussion

We found that heroin-dependent patients who joined MMT had a higher prevalence of heroin use during the MMT, with 31.0% of the subjects who had covertly used heroin in the past 6 months. A cross-sectional study of 14 MMT clinics in Guangdong province, China, reported that 68.7% of the subjects had used heroin in the past 30 days.^[15]^ The prevalence of this behavior was higher than that found in this study, possibly because Guangdong had a higher density of migrant workers and a higher immigration rate than Sichuan. The difficulty of obtaining MMT locally for non-residents made it possible for them to use heroin during MMT.

The study found that social capital theory may be useful in the design of relevant interventions, in that in the social capital theory module used in this study, social network, social support and social trust were significantly association with heroin use during MMT.

A negative correlation between the level of social networks and covertly using heroin during MMT, possibly because wider social networks might help people effectively handle life stress. As the origin of social capital, social network plays an important role in a relational society like China.^[31]^ A social network is a multi-dimension social connection. The network includes a variety of contacts, including innate network relations (relations with family members) and acquired network relations (relations with friends, colleagues or neighbors and other non-family members).^[34]^ It is an inevitable trend for everyone to develop his own resources and make use of social resources.^[35]^ We observed that the social network and the contact scope of heroin-dependent patients were very narrow (e.g., 28.3% of participants had a lot of close relationship in your life, 18.9% had usually been socializing with other people in the past month), which was consistent with the research results of Shen et al.^[29]^ One possible reason was that before MMT, in order to avoid arrest by police, heroin-dependent patients might try to hide their secrets.^[29]^ Another reason might be that heroin-dependent patients have tended to be self-enclosed and hide in order to avoid social discrimination.^[30]^

Heroin-dependent patients’ high level of social support was a protective factor for covertly using heroin during MMT, in that good social support might help heroin-dependent patients achieve higher socio-economic status (SES) driven by Chinese collectivism culture.^[31]^ Social support was closely associated with social network,^[36,37]^ which is also greatly influenced by the supports from family members. Good family support can not only provide material and other objective support, but also improve heroin-dependent patients’ confidence^[29]^ and self-support.^[38]^ Meanwhile, good social support might make heroin-dependent patients feel that they are not abandoned by the society and family,^[39]^ so as to reduce covertly using heroin during MMT and improve MMT compliance.^[40]^ Therefore, the focus of intervention on the families of heroin-dependent patients who joined MMT might effectively improve the treatment compliance of heroin-dependent patients.

Heroin-dependent patients’ high level of social trust was protective factor for covertly using heroin in the past 6 months. Social trust was the basis of social capital.^[41]^ Social trust was a kind of expectation based on morality, fairness, and interests in the context of certain knowledge and information.^[42]^ Chu et al^[43]^ found that heroin-dependent patients had a higher trust to medical professionals in MMT based on their desire to quit heroin in China. However, because they had been arrested by police before MMT, they were hostile to government, and their trust in the public security department were usually low.^[44]^Social trust might reflect institutional discrimination against heroin-dependent patients. Because of the risk of being caught while participating in MMT, heroin-dependent patients might be reluctant to go to the MMT clinic for MMT, which might affect their treatment compliance and may also lead to heroin use during the MMT.^[44]^

The study provided some insights for reducing covertly using heroin during MMT. First, social networks would improve access to social capital for heroin-dependent patients.^[36]^ China is an interpersonal society, and the relationships among people play an important role in social life, in that SES and social support often depend on the scale of social network.^[31]^ Heroin-dependent patients are encouraged to interact with network members normally and expand their social network actively. Meanwhile, governments can invest in infrastructure (e.g., mixed-income and mixed-use housing), as well as other environment (e.g., walkways) that provide opportunities for interaction between innate and acquired social networks of heroin-dependent patients.^[27]^ Second, good use of social support may reduce heroin use behavior of heroin-dependent patients during MMT.^[45]^ Through MMT-related knowledge training for family members of heroin-dependent patients, discrimination against heroin dependent patients can be eliminated, mutual trust can be established, and care and support for heroin dependent patients can be strengthened. At the same time, access to social support goes both ways.^[38]^ Heroin-dependent patients should also maintain an optimistic attitude and actively accept support from family members, friends, neighbors and colleagues. Third, we should find the importance of social trust in behavioral intervention. Social trust is an important indicator to connect heroin-dependent patients with society and institutions.^[41]^ To create a comfortable MMT environment for heroin-dependent patients and improve their social trust through establishing partnerships among medical and health institutions, government administration departments and social organizations and groups, and strengthening communication and collaboration.

Some limitations should be noted in this study. First of all, although the questionnaire is not a standardization questionnaire, it developed according to China's national conditions. In this study, the questionnaire showed good reliability and validity, the Cronbach alpha of the scales of social capital formed ranged from 0.71 to 0.92, as well as, the cumulative variance contribution rate of the items of social capital was 56.81%. Secondly, occurrence of covertly using heroin was self-reported and might subject to social preference desirability. However, we adopted and face-to-face methods to collect behavioral information of heroin dependent patients, and chose quiet places for investigation while ensuring the anonymity of the questionnaire, in order to reduce such bias. Thirdly, we considered including HIV infection as a comorbidity in the questionnaire. However, during the investigation, we could not collect the physical examination data of the participants. HIV infection status was only orally answered by the participants, with bias, so it was not included in the data analysis. Fourth, the study did not find a relationship between community participation and heroin use, possibly because community participation in the population was very low, which may reduce the statistical power to find the differences between groups. Fifth, our study only selected MMT clinics with a cumulative population of more than 1000 as sample objects. As the 49 MMT outpatient clinics in Sichuan province are scattered, and our project fund is limited, it is convenient for us to obtain more information we want by drawing more MMT outpatient clinics. However, such sampling may lose some valuable information and have selection bias. Finally, this is a cross-sectional study, which means it cannot be inferred causal relationships. Therefore, prospective studies are needed to confirm our findings.

## 5 Conclusions

The heroin-dependent patients’ social capital has a protective effect on covertly using heroin during MMT. Future interventions should make use of the social capital of heroin-dependent patients, expand their social networks, increase levels of social support, reduce social discrimination, and improve their trust in society.

## Acknowledgments

We thanks for the Center for AIDS/STD Control and Prevention, Sichuan Center for Disease Control and Prevention for supporting the data for analysis. We thanks for School of Public Health, Sun Yat-Sen University for providing methodological support. We also thanks for the students from West China School of Public Health for organizing the data for analysis.

## Author contributions

**Conceptualization:** Bo Gao, Peng Jia, Shujuan Yang.

**Data curation:** Bin Yu, Jiayu Han.

**Formal analysis:** Shifan Yang, Shujuan Yang.

**Funding acquisition:** Yi Gong.

**Investigation:** Shifan Yang, Bin Yu, Jiayu Han, Peijie Dong.

**Methodology:** Bo Gao, Jing Gu.

**Project administration:** Shujuan Yang.

**Resources:** Yi Gong.

**Supervision:** Jing Gu.

**Validation:** Bo Gao, Shujuan Yang.

**Writing – original draft:** Shifan Yang, Shujuan Yang.

**Writing – review & editing:** Shifan Yang, Bo Gao, Shujuan Yang.
